# CNS Expression of B7-H1 Regulates Pro-Inflammatory Cytokine Production and Alters Severity of Theiler's Virus-Induced Demyelinating Disease

**DOI:** 10.1371/journal.pone.0018548

**Published:** 2011-04-08

**Authors:** D'Anne S. Duncan, Stephen D. Miller

**Affiliations:** 1 Interdepartmental Neuroscience Program, Northwestern University Feinberg School of Medicine, Chicago, Illinois, United States of America; 2 Department of Microbiology-Immunology and Interdepartmental Immunobiology Center, Northwestern University Feinberg School of Medicine, Chicago, Illinois, United States of America; La Jolla Institute of Allergy and Immunology, United States of America

## Abstract

The CNS is a unique organ due to its limited capacity for immune surveillance. As macrophages of the CNS, microglia represent a population originally known for the ability to assist neuronal stability, are now appreciated for their role in initiating and regulating immune responses in the brain. Theiler's murine encephalomyelitis virus (TMEV)-induced demyelinating disease is a mouse model of multiple sclerosis (MS). In response to TMEV infection *in vitro*, microglia produce high levels of inflammatory cytokines and chemokines, and are efficient antigen-presenting cells (APCs) for activating CD4^+^ T cells. However, the regulatory function of microglia and other CNS-infiltrating APCs in response to TMEV *in vivo* remains unclear. Here we demonstrate that microglia increase expression of proliferating cell nuclear antigen (PCNA), and phenotypically express high levels of major histocompatibility complex (MHC)-Class I and II in response to acute infection with TMEV in SJL/J mice. Microglia increase expression of the inhibitory co-stimulatory molecule, B7-H1 as early as day 5 post-infection, while CNS-infiltrating CD11b^+^CD11c^−^CD45^HIGH^ monocytes/macrophages and CD11b^+^CD11c^+^CD45^HIGH^ dendritic cells upregulate expression of B7-H1 by day 3 post-infection. Utilizing a neutralizing antibody, we demonstrate that B7-H1 negatively regulates TMEV-specific *ex vivo* production of interferon (IFN)-γ, interleukin (IL)-17, IL-10, and IL-2 from CD4^+^ and CD8^+^ T cells. *In vivo* blockade of B7-H1 in SJL/J mice significantly exacerbates clinical disease symptoms during the chronic autoimmune stage of TMEV-IDD, but only has minimal effects on viral clearance. Collectively, these results suggest that CNS expression of B7-H1 regulates activation of TMEV-specific T cells, which affects protection against TMEV-IDD.

## Introduction

Central nervous system (CNS) infections are a challenge in host defense and effective pathogen clearance due to the lack of lymphatic drainage and minimal immune surveillance [1], [2]. It is well-established that CNS-resident microglia serve as a critical cell type for host defense, capable of initiating and regulating rapid responses against infections [2]. Due to their innate (cytokine and chemokine secretion) and adaptive (phagocytosis and T cell activation) immune functions, microglia serve as the first line of host defense in the CNS, necessary for recruitment and activation of infiltrating effector immune cells [3].

Theiler's murine encephalomyelitis virus-induced demyelinating disease (TMEV-IDD) is a virus-induced experimental mouse model of the human disease, multiple sclerosis [4]. TMEV is a single-stranded RNA virus, and intracranial inoculation of susceptible mouse strains (SJL/J) leads to the development of a chronic-progressive CD4^+^ T cell-mediated demyelinating disease [4], [5], [6]. As TMEV-IDD progresses, epitope spreading gives rise to CD4^+^ T cell responses against myelin peptides, proteolipid protein (PLP)_139–151_, PLP_56–70_, myelin oligodendrocyte glycoprotein (MOG_92–106_), PLP_178–191_, and myelin basic protein (MBP_84–104_) [5], [7]. TMEV persistently infects astrocytes, microglia, peripheral macrophages and dendritic cells [5], [6], [8], as well as stimulates microglial production of tumor necrosis factor (TNF-α), interleukin (IL)-6, IL-1β, IL-23p19, IL-12p70, CCL2, CCL3, CCL5, and CXCL10 *in vitro* [8], [9], [10]. Additionally, microglia and CD11b^+^F4/80^+^CD45^HIGH^ macrophages robustly increase cell surface expression of major histocompatibility complex (MHC) class I and MHC class II, costimulatory molecules (CD80, CD86, and CD40), and are efficient antigen presentation cells (APCs) stimulating proliferation and interferon (IFN)-γ secretion of virus-specific CD4^+^ T cells [9], [10], [11], [12]. These studies support a role of microglia and macrophages as a functional APCs in virus-induced inflammation and demyelination. However, the mechanism(s) by which CNS-resident and -infiltrating APCs regulate effector T cell responses in acute TMEV infection have yet to be explored.

Mechanisms exist between T cells and infected APCs, which elicit T cell dysfunction during viral infections. Negative co-stimulatory molecules, belonging to the B7:CD28 family, programmed death receptor-1 (PD-1) and its ligands, programmed death-ligand 1 (B7-H1/PD-L1) and programmed death-ligand 2 (B7-DC/PD-L2) inhibit effector T cell responses [13], [14]. B7-H1 is constitutively expressed on T and B cells, and is increased upon stimulation with interferon (IFN-γ) and IFN-β [15]. Microglia, astrocytes, oligodendrocytes, and neurons all express constitutive levels of B7-H1, which is upregulated upon IFN-γ stimulation [15], [16], [17], [18]. Signaling between B7-H1:PD-1 leads to an “exhausted” T response, characterized by decreased proliferative capacity, cytokine secretion, and cytotoxic effector functions [19]. Functional blockade of these interactions enables increased effector T cell function during viral infection [19], in contrast, in models of CNS autoimmunity, these interactions are reported to dampen autoreactive T cell responses protecting the CNS [15], [16].

To date, few studies have examined the function of the B7-H1:PD-1 pathway in CNS infections. Infection with the JHM strain of mouse hepatitis virus (JHMV), leads to transient expression of B7-H1 by microglia, but higher, sustained expression by oligodendrocytes; suggesting cell-type specific regulation of B7-H1 in CNS infections [17]. Additionally, neuronal expression of B7-H1 limits persistence of rabies virus, leading to protection against infection [18]. Thus, we hypothesized that B7-H1 signaling by microglia and other CNS-infiltrating APCs are critical for the regulation of virus specific effector T cell function, which may dampen the immune response to TMEV infection *in vivo*.

In this report, we describe phenotypic and effector functions of microglia in acute *in vivo* infection of SJL/J mice with TMEV. Microglia display differential expression patterns of antigen presentation molecules, I-A^s^, H-2K^s^, CD80, CD86, but express elevated and sustained levels of B7-H1 as early as day 3 post-infection compared to sham-infected microglia. CNS-infiltrating CD11b^+^CD11c^−^ monocytes/macrophages and CD11b^+^CD11c^+^ dendritic cells highly express B7-H1 compared to microglia following TMEV infection. *Ex vivo* blockade of B7-H1 on TMEV-infected mononuclear cells enhances IFN-γ/IL-17 and IFN-γ production from CD4^+^ and CD8^+^ T cells, respectively. Blockade of B7-H1 in virus-infected SJL/J mice leads enhanced severity of TMEV-IDD during the chronic autoimmune phase of disease, although mice that received anti-B7-H1 neutralizing antibodies have only slightly elevated virus levels in the brain. These data are the first to demonstrate a role for B7-H1 in regulating virus-specific effector T cell response in the CNS thereby limiting TMEV-IDD *in vivo*.

## Materials and Methods

### Mice

Female SJL/J mice (5–6 weeks) were purchased from Harlan Labs (Indianapolis, IN). All mice were housed under specific pathogen-free conditions in the Northwestern University CCM facility (Animal Welfare Assurance Number A3283-01) and experiments performed according to Protocol 2007-1285 approved by the Northwestern University Animal Care and Use Committee.

### Induction of TMEV-IDD

Mice were anesthetized with isoflurane (Bulter Animal Health Supply; Dublin, OH) and intracranially inoculated in the right cerebral hemisphere with 3×10^7^ plaque forming units (PFU) of the BeAn strain 8386 of TMEV in 30 µl serum-free Dulbecco's Modified Eagle's Medium (DMEM). Mice were examined two to three times per week for the development of chronic gait abnormalities and spastic paralysis indicative of demyelination, and assigned a clinical score of 0 to 6 as follows: 0 = asymptomatic, 1 = mild waddling gait, 2 = severe waddling gait, intact righting reflex, 3 = severe waddling gait, spastic hind limb paralysis, impaired righting reflex, 4 = severe waddling gait, spastic hind limb paralysis, impaired righting reflex, mild dehydration and/or malnutrition, 5 = total hind limb paralysis, severe dehydration and/or malnutrition, 6 = death.

### CNS Mononuclear Cell Isolation

Mice were anesthetized, transcardially perfused with phosphate buffered saline (PBS), and CNS cells were isolated as described previously [20], with the following adjustments. After digestion with DNAse I (Sigma Aldrich; St. Louis, MO) and Liberase (Roche Molecular Biochemicals; Indianapolis, IN), softened tissue was pushed through a 40 µM metal cell strainer.

### Flow Cytometry and Antibodies

Single cell suspensions for analysis were blocked with 10% rat serum, 10% mouse serum and antiCD16/32 for 20 minutes at 4°C before staining with multicolor antibody cocktails. Directly conjugated antibodies specific for mouse antigens CD3 (145-2C11; 1∶150), CD4 (GK1.5; 1∶150), CD8 (53-6.7; 1∶150), CD11b (M1/70; 1∶200), CD11c (N418; 1∶150), CD45 (30-F11; 1∶250), CD80 (16-10A1; 1∶150), CD86 (GL1; 1∶150), CD40 (HM40.3; 1∶150), H-2K (KH49; 1∶150), I-A (KH116; 1∶150), B7-H1 (MIH5; 1∶150), and PD-1 (J43; 1∶150) were purchased from BD Pharmingen or eBioscience (San Diego, CA). Proliferating cell nuclear antigen-FITC (PCNA) used at a 1∶100 dilution was purchased from DakoCytomation. Biotinylated anti-I-A^s^ (MKS4) was labeled with streptavidin-PeCy7 (eBioscience). Isotype antibodies for individual staining antibodies were used to define positive and negative staining. Data were acquired on a FACS CANTO cytometer (BD; Sparks, MD) and analyzed using Flow Jo (Treestar; Ashland, OR) software.

### Neutralization of B7-H1 Signaling

CNS mononuclear cells were isolated from sham- and TMEV-infected tissue. Bulk cells were seeded at a cell density of 1×10^5^/well and co-cultured in the presence of 10 µM Rat IgG2a (isotype control) or anti-B7H1 monoclonal Ab (mAb; clone MIH5, eBioscience, San Diego, CA) and 0 or 50 µM VP2_70–86_ (CD4^+^ immundominant peptide: WTTSQEAFSHIRIPLPH) or VP3_159–166_ (CD8^+^ immundominant peptide: FNFTAPFI; Genemed Synthesis, Inc., South San Francisco, CA). Cells were cultured in RPMI (Sigma Aldrich, St. Louis, MO) with 10% fetal bovine serum (FBS), 2 mM L-glutamine (Life Technologies, Gaithersburg, MD), 100 U/mL penicillin (Life Technologies), 100 mg/mL streptomycin (Life Technologies), 0.1 mM non-essential amino acids (Sigma Aldrich), 50 mM 2-mercaptoethanol, 25 mM HEPES (Mediatech, Masnassas, VA), 1 mM aminoguanidine (Sigma Aldrich) and 20 mM indomethacin (Sigma Aldrich) to suppress nitric oxide synthetase and prostaglandin production. Cultures of CNS cells alone and CNS cells stimulated with αCD3 (Clone 2C11; Bio X Cell; West Lebanon, NH) served as negative and positive controls, respectively. Following 72-hour incubation at 37°C, culture supernatants were collected and stored at −20°C until analysis. Cytokines and chemokines (IL-2, IFNγ, IL-17, IL-10) were measured from cultured supernatants and analyzed using the CBA Cell Signaling Flex Kit (BD Biosciences). Data acquisition from samples was collected using a FACS CANTO cytometer and analyzed using the FCAP Array Software (BD Biosciences).

### *In vivo* Blockade of B7-H1

To determine the function of B7-H1 on the development and severity of TMEV-IDD *in vivo*, female SJL/J mice were administered two doses (100 µg/mouse) of Rat IgG2b (clone 2A3; Bio X Cell, West Lebanon, NH) or αB7-H1 (clone 10F.9G2; Bio X Cell, West Lebanon, NH) at days 0 and ^+^3 (relative to infection) and monitored for clinical signs of disease.

### TMEV plaque assay

Brains and spinal cords from Rat IgG2a and αB7-H1 treated mice at days 7 and 14 post-infection were collected from anesthetized mice, weighed, and homogenized on ice using a Polytron System PT1200C tissue homogenizer (Kinematica AG, Switzerland). CNS homogenates were serially diluted and added to tissue culture-treated plates (Nunc, Roskilde, Denmark) of confluent BHK-21 cells (American Type Culture Collection, Manassas, VA) for a 1 h incubation at room temperature, with periodic gentle rocking. A media/agar solution containing 1% Noble Agar (BD, Sparks, MD) and 2× DMEM was added to cells after the 1 h incubation. Following a 5 day incubation at 34°C, formalin (Fisher Scientific, Fair Lawn, NJ) was added and incubated at room temperature for 1 h to fix the BHK monolayer. The agar was removed from the plates and plaques were visualized by staining with crystal violet. To determine PFU/ml homogenate, the number of plaques on each plate was multiplied by the dilution factor of the homogenate and divided by the amount of homogenate added per plate. The PFU/ml was divided by the weight of the tissue to calculate PFU/g tissue.

### Statistical Analyses

Comparisons were performed using two-tailed Student's *t*-test. Comparisons of more than two groups were analyzed using a two-way ANOVA. Two-way ANOVA with a Bonferroni post-test was used to determine statistical differences between mean clinical disease scores.

## Results

### TMEV infection stimulates an increase in microglia cell numbers and PCNA expression

**Figure 1:**
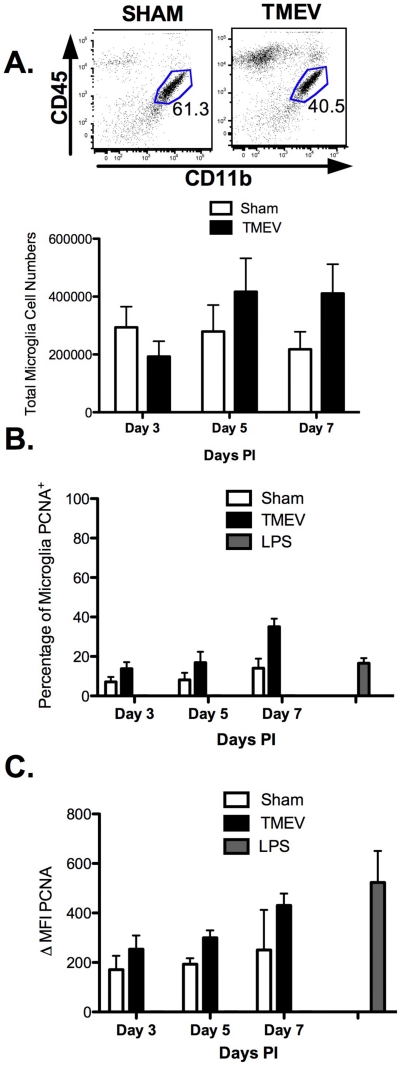
TMEV infection induces increased microglia cell numbers associated with elevated levels of PCNA expression. **A**. Microglia were isolated from sham-infected and TMEV-infected SJL/J mice at day 3, 5, and 7 post-infection and identified by CD11b and CD45 expression via flow cytometric analysis using the illustrated gating strategy. The data is representative of 2–3 independent experiments with an average of 3–5 mice per group per experiment. **B**. The percentage of cells expression Proliferation Cell Nuclear Antigen (PCNA) was measured in microglia (CD11b^+^CD45^LOW^) isolated during the acute phase of TMEV infection using flow cytometry. **C**. Mean Fluorescence Intensity (MFI) of PCNA expression in microglia isolated from TMEV-infected mice compared to sham-infected mice. Microglia isolated from LPS-treated mice (200 µg/for 15 hours) served as a positive control. These data are representative of four independent experiments with n = 4–5/group expressed as the average ± SEM.

The contribution of microglia to initiate CNS innate and adaptive immune responses to TMEV infection has been extensively studied *in vitro* [8], [9]. Further, our laboratory has previously demonstrated that microglia display a similar phenotype and function to CNS-infiltrating macrophages during chronic TMEV-IDD *in vivo* [10]. To further study the function of this cell type during the innate *in vivo* antiviral responses against TMEV, we examined immune functions of microglia in acute TMEV infection. We first quantified the total number of microglia from the brains and spinal cords of sham- and TMEV-infected at days 3, 5, and 7 post-infection. As shown in **Figure 1A**, TMEV infection induced increased microglia cell numbers as early as day 5 post-infection, and the numbers remained elevated at day 7 post-infection. Thus, acute TMEV infection enhances the expansion of microglia *in vivo*.

We next determined a mechanism of microglial cell expansion following Theiler's virus infection. Murine microglia undergo rapid proliferation and migrate to sites of CNS injury and infection *in vivo*. To quantify the proliferation, we examined the expression of proliferating cell nuclear antigen (PCNA), an intracellular protein that is abundant in all cells and accumulates in the S phase of the cell cycle [21], using flow cytometry. Expression of PCNA by microglia isolated from sham- and TMEV-infected mice was analyzed at days 3, 5, and 7 post-infection. As demonstrated in **Figure 1B**, microglia upregulate PCNA expression as early as day 3 post-infection compared to microglia from sham-infected mice (13.75±3.35% vs. 7.13±2.47%) with the most significant increase observed at day 7 post-infection (35.07±2.56% vs. 14.05±4.85%). The mean fluorescence intensity also correlates with the overall increased percentages of PCNA^+^ microglia, with the most significant increase at day 7 (430.33±47.70 vs. 250.50±161.50) (**Figure 1C**). These data indicate that microglia respond rapidly to TMEV infection by increasing in number and expression of PCNA.

### Microglia and CNS-Infiltrating APCs express differential levels of antigen presentation molecules after TMEV infection

**Figure 2:**
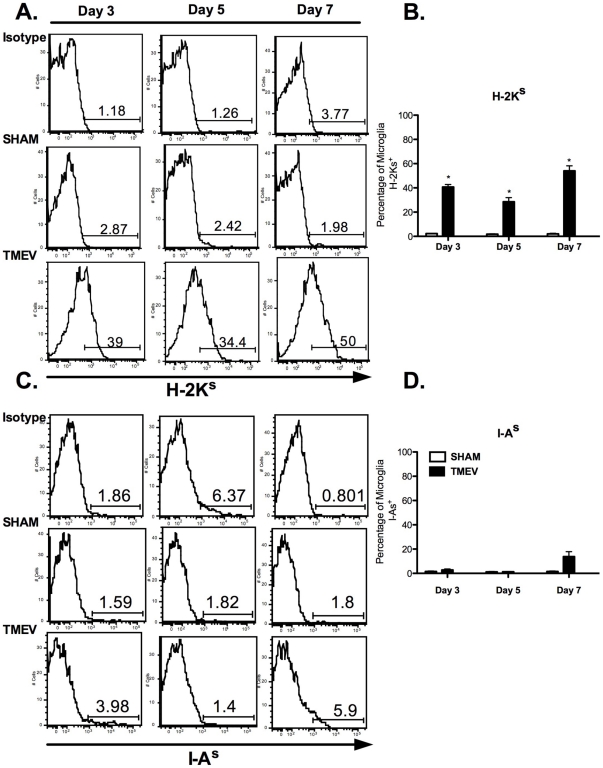
TMEV induces differential expression of antigen presentation molecules on microglia *in vivo*. **A**. Microglia (CD11b^+^CD45^LOW^) from sham- and TMEV-infected mice at days 3, 5, and 7 post-infection and analyzed for H-2K^s^ and I-A^s^ expression using flow cytometry. **A&B**. Histograms are representative of H-2K^s^ expression. **B**. Quantification of percent positive for H-2K^s^ are reflected based of negative staining from isotype controls and sham-infected microglia. **C&D**. Histograms are representative of I-A^s^ expression. Quantification of percent positive for I-A^s^ are reflected based of negative staining from sham-infected microglia. The data is representative of three independent experiments with n = 4–5 mice/group expressed as the average ± SEM. *p<0.05 compared to sham-infected mice.

Microglia constitutively express negative to low levels of B7-1 (CD80), B7-2 (CD86), and major histocompatibility complex (MHC) molecules. Expression of these molecules is upregulated in response to inflammatory and TMEV virus stimuli *in vitro* and *ex vivo* [9], [11], [12]. Additionally, the negative costimulatory molecule, B7-H1, is constitutively expressed by microglia and is increased following stimulation with IFN-γ and IFN-β [15], [16], [17], [18]. Therefore, we examined the expression of antigen presentation-related molecules by microglia following acute TMEV infection *in vivo*. Microglia were isolated from TMEV- and sham-infected mice and assessed for the expression levels of MHC class I (H-2K^s^), MHC Class II (I-A^s^), CD80, CD86, and B7-H1 using flow cytometry. Similar to previous *in vitro* findings, the percentage of microglia from TMEV-infected mice upregulating MHC class I expression was significantly greater than those from sham-infected mice at all time-points post-infection reaching 54.03±4.184% by day 7 post-infection (**Figure 2A&B**). In contrast to MHC class I, MHC Class II expression by TMEV-infected microglia was significantly delayed, as enhanced expression was only evident at day 7 post-infection (13.8±4.038%) compared to sham-infected microglia (1.55±0.431%) (**Figure 2C&D**).

**Figure 3:**
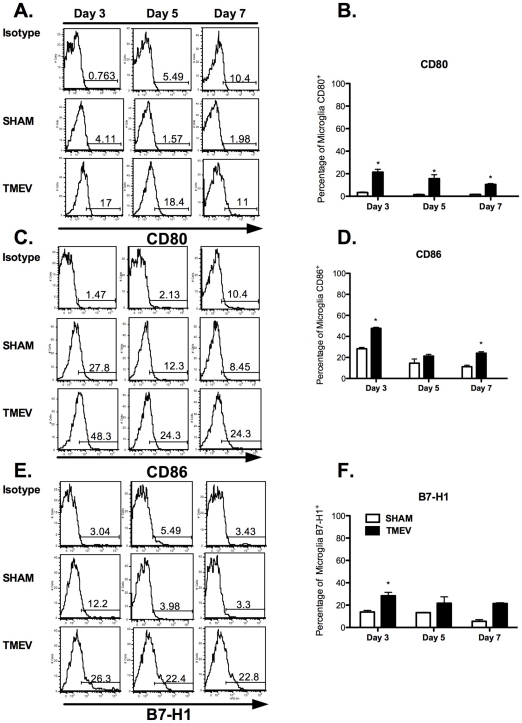
TMEV induces differential expression of positive and negative co-stimulatory molecules on microglia *in vivo*. **A**. Microglia (CD11b^+^CD45^LOW^) from sham- and TMEV-infected mice at days 3, 5, and 7 post-infection were analyzed for CD80, CD86, and B7-H1 expression using flow cytometry. **A&B**. Histograms are representative of CD80 expression. **C&D**. Histograms are representative of CD86 expression. **E&F**. Histograms are representative of B7-H1 expression. The data is representative of three independent experiments with n = 4–5 mice/group expressed as the average ± SEM. *p<0.05 compared to sham-infected mice.

**Figure 4:**
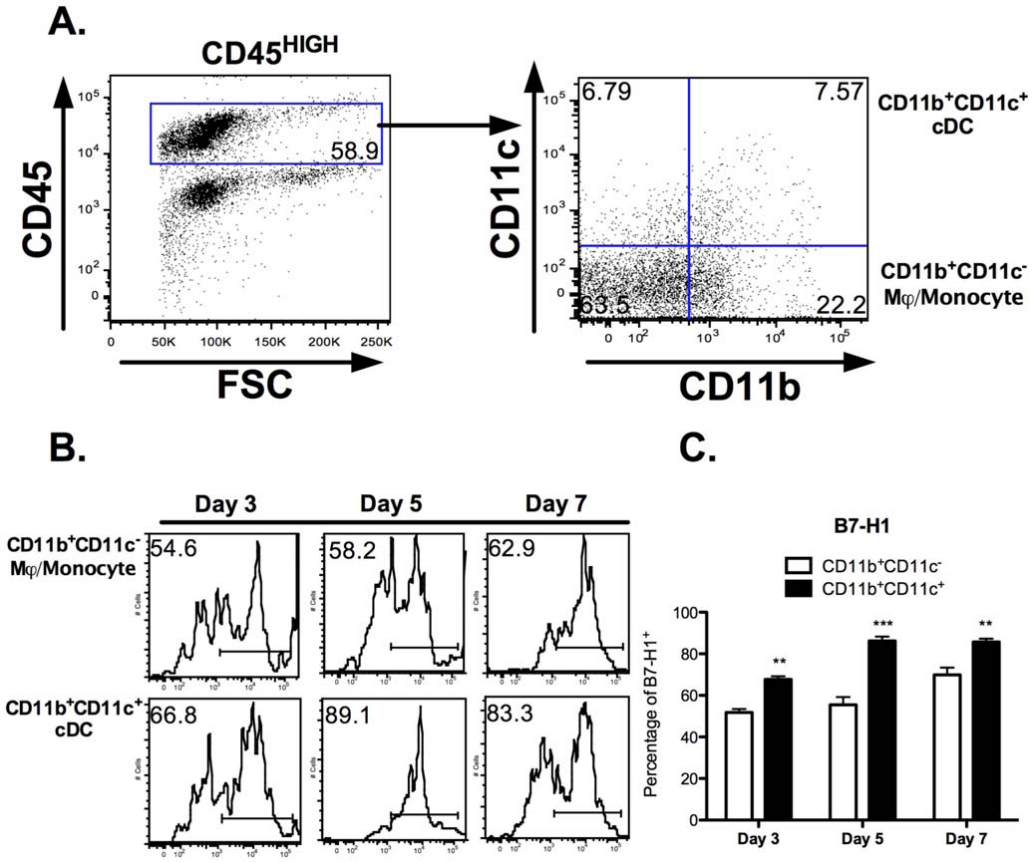
Expression of B7-H1 by CNS-Infiltrating APCs following TMEV infection *in vivo*. CNS-infiltrating APCs from TMEV-infected mice at days 3, 5 and 7 post-infection were analyzed for B7-H1 expression. **A.** Gating strategy identifying CD11b^+^CD11c^−^ monocytes/macrophages and CD11b^+^CD11c^+^ dendritic cells based on CD45^HIGH^ expression to exclude microglia. Numbers reflect total percentages of each cell population. **B.** Histograms of B7-H1 expression on monocyte/macrophage and dendritic cell populations following infection. **C.** Quantification of percent positive for B7-H1 are reflected based of negative staining from isotype controls and sham-infected cell populations. Black bars represent CD11b^+^CD11c^−^ monocytes/macrophages, while white bars reflect CD11b^+^CD11c^+^ dendritic cells. The data is representative of three independent experiments with n = 4–5 mice/group expressed as the average ± SEM. **p<0.01, ***p<0.001 compared to CD11b^+^CD11c^−^ infiltrating populations.

The inducible expression of positive and negative costimulatory molecules (CD80, CD86, B7-H1) by microglia was also examined following infection to gain further insight into their function during acute TMEV infection *in vivo*. TMEV significantly induces CD80 expression on microglia peaking at day 3 post-infection (21.40±2.57% vs. 3.4±0.37%) (**Figure 3A&B**). CD86 expression was similarly upregulated (**Figure 3C&D**). The differential expression of B7-H1 on microglia in response to inflammation [15], [16] raised the possibility that B7-H1:PD-1 interactions may play a pivotal role in regulating the initiation and duration of anti-TMEV responses *in vivo*. Therefore, we examined the expression patterns of B7-H1 by microglia from uninfected and TMEV-infected mice. Approximately 25% of microglia from TMEV-infected mice expressed are B7-H1^+^ within 3 days post-infection and the increased expression remained at day 7 post-infection (**Figure 3E&F**). In terms of the expression of B7-H1 by CNS-infiltrating CD45^HIGH^CD11b^+^CD11c^−^ macrophages and monocytes or CD45^HIGH^CD11b^+^CD11c^+^ conventional dendritic cells, CD11b^+^CD11c^+^ cells displayed significant enhanced expression of B7-H1 (**Day 3**: 67.733±1.462%; **Day 5**: 86.233±2.034%; **Day 7**: 85.700±1.550%) compared to CD11b^+^CD11c^−^ (**Day 3**: 51.8±1.677%; **Day 5**: 55.433±3.811%; **Day 7**: 69.833±3.524%) cells at all time-points following acute TMEV infection (**Figure 4A–C**). Consistent with previous findings [15], expression of B7-DC (B7-H2, a secondary ligand for PD-1) by microglia was undetectable, while expression was restricted to dendritic cells and macrophages following TMEV infection (data not shown). These data suggest microglia and CNS-infiltrating CD11b^+^CD11c^+/−^ APCs may be critical for effector T cell regulation due to elevated and sustained levels of B7-H1 during acute CNS encephalitis induced by TMEV infection.

### CD8^+^ T cells express high levels of B7-H1 and PD-1 in response to TMEV infection *in vivo*

**Figure 5:**
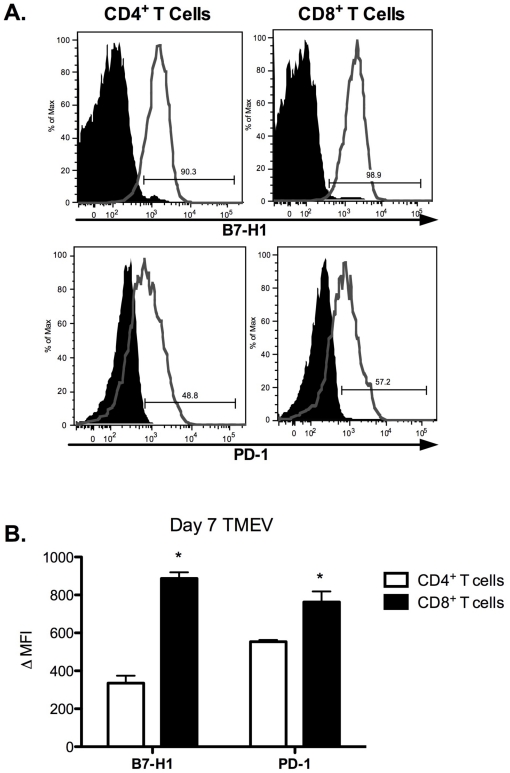
B7-H1 and PD-1 expression are highly upregulated on CD4^+^ and CD8^+^ CNS T cells from TMEV-infected SJL/J mice. CNS T cells from wild-type SJL/J mice at day 7 post-TMEV infection were analyzed for expression of B7-H1 and PD-1 molecules by flow cytometry. **A**. Histograms of B7-H1 and PD-1 expression on CD4^+^ and CD8^+^ T cells. Black shaded area represents isotype control and the grey line represents positive staining. A representative histogram from one individual mouse from three separate experiments is shown. **B**. Data is also represented as Δ mean fluorescence intensity (ΔMFI) of B7-H1 and PD-1 expression on CD4^+^ and CD8^+^ T cells. Expression of B7-H1 and PD-1 is significant greater (*p<0.05) on with CD8^+^ as compared to CD4^+^ T cells. Data are expressed as the average ± SEM.

Naïve CD4^+^ and CD8^+^ T cells express constitutive low levels of B7-H1, but expression remains high during chronic viral infections [19]. Activated T cells and “exhausted” CD8^+^ T cells express PD-1 [19]. B7-H1 and PD-1 expression was assessed on CD4^+^ and CD8^+^ T cells isolated from the CNS to determine their potential role in regulation of CNS-infiltrating effector T cell function at the site of infection. Both CD4^+^ and CD8^+^ T cells express similar levels of B7-H1 at day 7 post-infection (**Figure 5A**) (90.3%^+^ vs. 98.9%^+^), while CD8^+^ T cells express elevated levels of PD-1 represented by percent positive (48.8%^+^ vs. 57.2%^+^) and mean fluorescence intensity (**Figure 5B**) (**B7-H1:** 335.40±39.451 [CD4^+^ T cells] vs. 886.8±32.772 [CD8^+^ T cells]; (**PD-1:** 553.6±8.992 [CD4^+^ T cells] vs. 363.30±55.310 [CD8^+^ T cells]). The expression of B7-H1 and PD-1 on T cells decreased by day 14 post-infection (data not shown). These data highlight a potential mechanism by which CD8^+^ T cells can be impaired to perform effector functions, such as IFN-γ production, leading to an ineffective immune response to TMEV-IDD allowing the establishment of a persistent CNS TMEV infection.

### B7-H1 signaling regulates TMEV-specific CNS CD4^+^ and CD8^+^ T cell effector function

**Figure 6:**
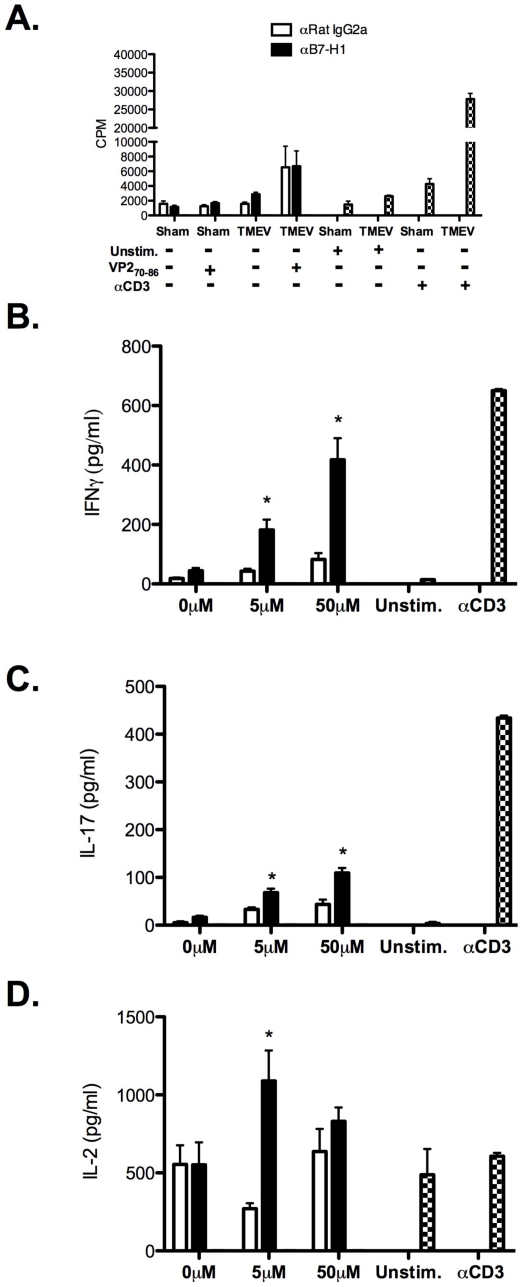
Blockade of B7-H1 enhances cytokine production from TMEV-specific CNS-infiltrating CD4^+^ T cells *ex vivo*. CNS (brain and spinal cord) mononuclear cells were isolated from TMEV-infected mice (n = 10/group) at day 7 post-infection and co-cultured in the presence of the CD4^+^ T cell restricted peptide-VP2_70–86_. Control Ig (open bars) and anti-mouse B7-H1 (black bars) were added to the cultures. Unstimulated and anti-mouse CD3-stimulated CNS mononuclear cells served as negative and positive controls, respectively. **A**. Proliferation was analyzed by ^3^[H]-TdR uptake of triplicate wells per group. **B–D**. After 72 h, supernatants from triplicate wells of each group in (A), the levels of IFN-γ (**B**), IL-17 (**C**), and IL-2 (**D**) were measured using CBA technology. Data are representative of three separate experiments. *p<0.05 compared to cultures containing rat IgG2a control.

**Figure 7:**
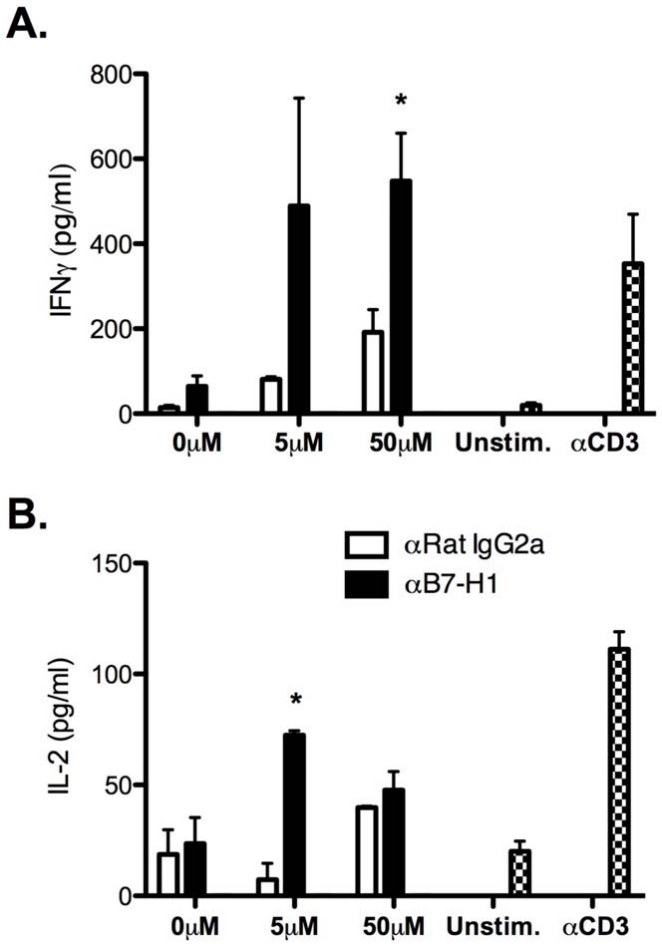
Blockade of B7-H1 enhances cytokine production from TMEV-specific CNS-infiltrating CD8^+^ T cells *ex vivo*. CNS (brain and spinal cord) mononuclear cells were isolated from TMEV-infected mice (n = 10/group) at day 7 post-infection and co-cultured in the presence of increasing concentrations of the CD8^+^ T cell restricted peptide-VP3_159–66_. Control Ig (open bars) and anti-mouse B7-H1 (black bars) were added to the cultures. Unstimulated and anti-mouse CD3-stimulated CNS mononuclear cells served as negative and positive controls, respectively. After 72 h, levels of IFN-γ (**A**), and IL-2 (**B**) were measured using CBA technology from the supernatants of triplicate wells. Data are representative of three separate experiments. *p<0.05 compared to cultures containing Rat IgG2a control.

PD-1:B7-H1 interactions inhibit T cell activation [14], [22], ultimately resulting in decreased proliferation and effector cytokine production *i.e.* IFN-γ [19]. To determine if B7-H1 signaling hampers CD4^+^ T cell function, CNS mononuclear cells isolated from sham- and TMEV-infected mice were stimulated in the absence or presence of VP2_70–86_ and anti-B7-H1 monoclonal antibody (mAb). Blockade of B7-H1 signaling did not affect the proliferation of CNS-infiltrating VP2_70–86_ CD4^+^ T cells (**Figure 6A**). However inhibition of B7-H1 signaling led to increased production of IL-17, IFNγ, and IL-2 in a VP2_70–86_ dose-dependent manner (**Figure 6B–D**). To determine if B7-H1 signaling down-regulates CD8^+^ T cell function, CNS mononuclear cells isolated from sham- and TMEV-infected mice were stimulated in the absence or presence of VP3_159–166_ and anti-B7-H1 mAb. Similar to CD4^+^ T cell responses, inhibition of B7-H1:PD-1 signaling enhanced production of IFN-γ and IL-2 in a VP3_159–166_ dose-dependent manner (**Figure 7A&B**). Unlike CD4^+^ T cells, we did not observe production of IL-17 from VP3_159–166_-stimulated CD8^+^ T cells following treatment with isotype or anti-B7-H1 (data not shown). These data demonstrate that B7-H1:PD-1 signaling inhibits effector cytokine production by CNS-infiltrating CD4^+^ and CD8^+^ T cells, further supporting a regulatory function of this receptor:ligand pair in controlling T cell effector functions against TMEV infection *in vivo*.

### B7-H1 negatively regulates the progression of chronic TMEV-IDD

**Figure 8:**
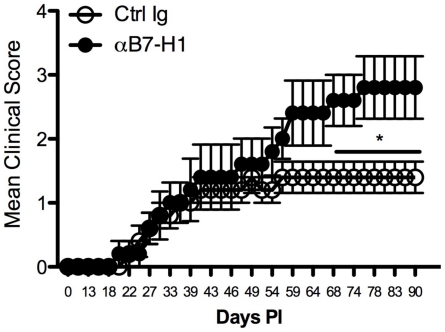
*In vivo* neutralization of B7-H1 enhances clinical disease in chronic TMEV-IDD. Wild-type SJL/J mice were infected with TMEV and monitored for clinical signs of disease until day 90 post infection following 100 µg/mouse injections of αRat IgG2b isotype control (○) and anti-B7-H1 mAb (•) at days 0 and +3 relative to infection. The x-axis represents days post-infection, while the y-axis represents the mean clinical score as defined in the materials and methods. The data is representative of 3 independent experiments with n = 5 mice/group expressed as the average ± SEM. *p<0.05 compared to mice receiving Rat IgG2b isotype control at each time point, 2-way ANOVA.

**Figure 9:**
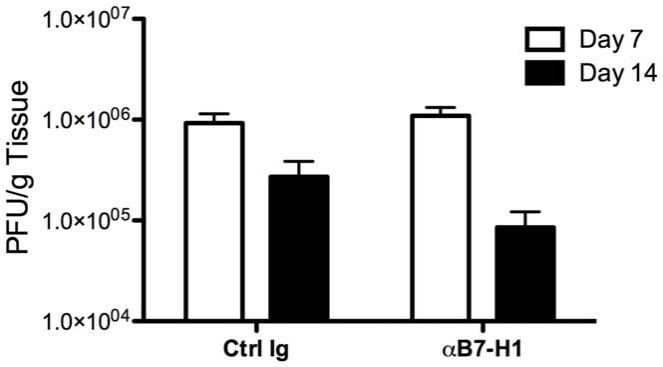
B7-H1 limits viral replication in TMEV infection. **A**. TMEV viral loads in the brain following treatment with anti-B7-H1 mAb or isotype control at days 7 and 14 post-infection. Data are expressed as plaque forming units/gram of tissue (PFU/g). The x-axis represents days post-infection, while the y-axis represents PFU/g tissue. The data is representative of 2 independent experiments with n = 2–4/group expressed as the average ± SEM.

Elevated and sustained levels of B7-H1 by CNS-resident microglia and CNS-infiltrating T cells support a role for B7-H1:PD-1 signaling in controlling TMEV viral clearance and potentially regulating onset and progression of TMEV-IDD *in vivo*. To further support this hypothesis, the development of TMEV-IDD was compared in anti-B7-H1 mAb-treated and control Ig-treated control mice. The onset of clinical symptoms of TMEV-IDD was comparable in anti-B7-H1 mAb and control mice (**Figure 8**), however the severity of clinical disease was significantly increased in the chronic phase of disease (>day 55–60 post-infection) and this was also reflected by histopathological examination of inflammation and demyelination (data not shown). Viral clearance of TMEV was also assessed following treatment with anti-B7-H1 mAb. TMEV viral loads in the brain remained similar between anti-B7-H1 mAb and control treated mice at day 7 and day 14 post-infection (**Figure 9**). Although B7-H1 and PD-1 expression is observed on CNS-resident microglia and CNS-infiltrating T cells, anti-B7-H1 mAb treatment does not significantly alter the viral clearance and onset of disease, but does increase the severity of TMEV-IDD *in vivo* indicating an important role for this molecule in regulating the chronic autoimmune phase of disease.

## Discussion

CNS infections represent a unique challenge to the host due to the relative paucity of surveillance by peripheral immune cells. Thus, understanding the immune functions of microglia and other CNS-resident cells during CNS infections is critical for promoting host defense and clearance of the infection [1], [2]. Infection with TMEV, a single-stranded RNA virus, leads to a chronic progressive demyelinating disease in susceptible mouse strains associated with persistent CNS virus infection and serves as an relevant experimental mouse model of MS. In the present study, we examined the effector functions of microglia in response to acute TMEV infection *in vivo*, and focused on understanding a potential mechanism by which the negative co-stimulatory molecule B7-H1 regulates virus-specific T cell responses and may contribute to the induction of chronic autoimmune demyelination.

We have described temporal and phenotypic characteristics of microglia after TMEV infection. Microglia cell numbers increase over time in response to TMEV infection *in vivo* (**Figure 1**), suggesting these cells are the first responders cells with the ability to proliferate and migrate to the site of infection [3]. Due to the increase in microglial cell numbers following TMEV infection, we hypothesized that microglia were proliferating *in situ* or a peripheral monocyte/macrophage population may be contributing to the overall increase in cell numbers. Using flow cytometric analyses, microglia increase expression of the nuclear cell-cycle protein, PCNA in response to TMEV infection with peak expression observed at day 7 post-infection (**Figure 1**). To distinguish between a peripheral immune cell population infiltrating and differentiating into microglia after TMEV infection, we also examined the expression of Ly6C, a marker of “inflammatory monocytes” using flow cytometry [23]. Our results indicate that microglia were Ly6C^LOW^ suggesting that microglia were proliferating in the CNS, and a peripheral monocyte/macrophage population was not contributing to the microglia pool in TMEV-IDD *in vivo* (data not shown).

Our laboratory has extensively studied the innate immune functions of neonatal microglia *in vitro*. Upon TMEV infection, neonatal microglia produce high levels of TNF-α, IL-6, IFN-α/β, IL-12p40 compared to uninfected microglia *in vitro* [8], [9]. Additionally, microglia upregulate surface expression of MHC Class I and MHC Class II, and gain the ability to stimulate production of IFN-γ from TMEV-specific CD4^+^ T cells [8], [9]. However, the focus of this study was to examine the functions of microglia isolated from adult mice *in vivo*, since microglia isolated from neonatal and adult mice display differential responses to antigens [24]. In comparison to previous *in vitro* studies, our data show that microglia display increased levels of MHC Class I as early as day 3 post-infection, while increased MHC Class II levels are only most evident at day 7 post-infection *in vivo* (**Figure 2**). Consistent with other studies, these data highlight the differential regulation of MHC Class I and II expression on microglia during acute TMEV infection [25], and potentially provide insight into how these cells may contribute to the reactivation of CNS-infiltrating CD4^+^ and CD8^+^ T cells after TMEV infection *in vivo*. Microglia also display differences in CD80 and CD86 expression following TMEV infection *in vivo* (**Figure 3**), which differs from the expression patterns observed with our *in vitro* studies in that upregulation is delayed. Our data also indicate that microglia express elevated and sustained levels of B7-H1 following TMEV infection, suggesting these cells may be critical for negatively affecting antiviral T cells responses [15], [17], [18]. Consistent with previous findings, increased expression of MHC Class I and slightly elevated B7-H1 expression by microglia after acute TMEV infection, suggest that microglia may not be the dominant cell type contributing to the control of CD8^+^ T cell effector function [17]. It can be hypothesized that CD45^HIGH^CD11b^+^CD11c^−^ CNS-infiltrating monocytes/macrophages may be more critical for the regulation of CD8^+^ T cell effector function as measured by significantly higher expression of both MHC Class I and B7-H1 compared to microglia (**Figure 4**). The possibility of B7-H1:CD80 interactions limiting CD4^+^ T cell proliferation and cytokine secretion has been previously reported [26] and is outside the scope of our studies. However, it cannot be excluded as an alternative APC:T cell interaction, contributing to antiviral mechanisms in TMEV clearance.

Activated T cells express high levels of B7-H1 and PD-1 [14], [27]. During TMEV infection, CNS-infiltrating CD4^+^ and CD8^+^ T cells express increased levels of B7-H1 and PD-1 at day 7 post-infection, though CD8^+^ T cells display significantly higher levels (**Figure 5**). Due to enhanced B7-H1 expression in response to TMEV infection by CNS-resident and -infiltrating APCs and T cells, we examined a potential mechanism of how this molecule may regulate cytokine production in our infection model. Our results indicate that blockade of B7-H1 enhances the production of IFN-γ/IL-17/IL-2 by CD4^+^-restricted (VP2_70–86_) and IFN-γ/IL-2 by CD8^+^-restricted (VP3_159–66_) T cell responses *ex vivo* (**Figures 6** **and** **7**). Although it has been described that B7-H1 expression is critical for myeloid DC (CD45^HIGH^CD11b^+^CD11c^+^) regulation of IFN-γ and IL-17 production during autoimmunity [16]; based on our findings, it remains unclear which APC (microglia, macrophages, or subsets of DCs) populations is/are most dominant for regulating CD4^+^ and CD8^+^ T cell responses through B7-H1:PD-1 or B7-DC:PD-1 interactions during acute and chronic TMEV-IDD *in vivo*.

To date, the functional consequences of B7-H1 inhibition have been extensively explored in other models of virus-induced demyelination, such as infection with the JHMV strain of mouse hepatitis virus (MHV) [17]. Inhibition of B7-H1 significantly enhances the clinical course of JHMV infection *in vivo*, without altering demyelination. Our data indicate that blockade of B7-H1 did not have a significant effect on disease onset, but enhanced the chronic course of TMEV-IDD during the autoimmune phase of disease (**Figure 8**), although mice treated with anti-B7-H1 mAb have similar TMEV viral loads in the brains at day 7 post-infection compared to isotype controls (**Figure 9**). Although our data do not support previous findings that inhibition of B7-H1:PD-1 signaling positively affects viral clearance, our *in vivo* results suggests that B7-H1 plays a protective role during the autoimmune phase of TMEV-IDD perhaps by limiting epitope spreading to myelin epitopes. Our results are supported by evidence in the experimental autoimmune encephalomyelitis (EAE) model of MS, which indicates that B7-H1:PD-1 interactions are necessary for protection against autoimmunity. Evidence for this conclusion includes: (a) B7-H1^−/−^ mice or mice receiving neutralizing αB7-H1 mAb are hyper-susceptible to PLP_139–151_-induced relapsing remitting EAE in SJL mice [15], [16]; (b) adoptive transfer of wild-type encephalitogenic T cells into B7-H1^−/−^ recipients results in enhanced severity of MOG_35–55_-induced EAE disease in C57BL/6 mice [28]; (c) blockade of PD-1 using a neutralizing monoclonal antibody or induction of MOG_35–55_-induced EAE in PD-1^−/−^ mice leads to enhanced disease [29], [30]; and (d) CNS-resident microglia express constitutive and inducible levels of B7-H1 *in vitro* and *in vivo* compared to astrocytes and splenocytes, suggesting these cells are critical for regulation of T cell responses during infection and autoimmunity [15]. During TMEV infection, neutralization of B7-H1 affects the production of IFN-γ, IL-17, and IL-2 from CD4^+^ T cells and IFN-γ, IL-10, IL-2 from CD8^+^ T cells *ex vivo* and the substantial increase in production of these cytokines does lead to a substantial protective effect on TMEV-IDD development in the absence of B7-H1 signaling.

In summary, our data support the conclusion that CNS expression of B7-H1 by CNS APCs, including microglia and infiltrating CD11b^+^CD11c^−^ and CD11b^+^CD11c^+^ cells as well as B7-H1 and PD-1 expression by CNS-infiltrating T cells regulates production of IFN-γ, IL-17, and IL-2. In terms of development of TMEV-IDD, *in vivo* monoclonal antibody blockade of B7-H1:PD-1 signaling does not significantly alter the onset of disease, only the severity observed during the autoimmune phase. These findings support the idea that B7-H1:PD-1 signaling may serve as a potential mechanism that regulates APC:T cell interactions affecting the progression of virus-induced demyelination and autoimmunity.
